# Prevention of unplanned extubation in neonates with silk tie securement

**DOI:** 10.1038/s41390-025-04168-w

**Published:** 2025-06-12

**Authors:** Tetyana H. Nesterenko, Firas Saker, Daniel Kubiak, John Dickson, Amanda L. Sheppard, Nicole Saliba, Katherine L. Fedor, Mohamed A. Mohamed, Hany Aly

**Affiliations:** 1 Neonatology, Cleveland Clinic Children’s Hospital, Cleveland, OH USA; 2 Continuous Improvement, Cleveland Clinic, Cleveland, OH USA

## Abstract

**Background:**

Unplanned extubation (UE) is the fourth most common adverse event in neonatal intensive care units (NICUs) and remains a significant global challenge. Standardizing endotracheal tube (ETT) maintenance could reduce UE rates to 0.5–1.0 events per 100 non-tracheostomy ventilation days. This quality improvement (QI) project aimed to reduce the UE rate to below 1.0 event per 100 non-tracheostomy ventilation days within 12 months.

**Methods:**

Using a QI methodology, we conducted four Plan–Do–Study–Act (PDSA) cycles, including frequent ETT securement evaluations, ETT stabilization during procedures, audits of chest radiographs, and the use of a silk tie for ETT reinforcement. Statistical process control charts monitored progress.

**Results:**

Baseline UE rate was 1.73 events per 100 non-tracheostomy ventilation days. The first three PDSA cycles (January 2019–May 2020) produced a non-significant signal for reduced UE to 0.88. After introducing the silk suture tie in PDSA cycle 4, the rate significantly declined to 0.58 and was sustained from October 2020 to December 2021. A further special cause variation occurred from February to December 2022 with UE reduction to 0.06.

**Conclusions:**

A multidisciplinary approach and silk suture tie intervention significantly reduced and sustained one of the lowest reported UE rates, enhancing ETT securement and patient safety.

**Impact:**

- Unplanned extubation (UE) events are not uncommon in the neonatal intensive care unit.
- This project demonstrates that the lowest reported UE rates in neonatal ICUs are possible without incurring additional costs or requiring new securement devices.
- The success of this project underscores the importance of standardizing care, reducing variability, and fostering a multidisciplinary, collaborative approach.
- The silk tie enhancement for ETT stabilization offers a practical, scalable solution to prevent UEs and improve neonatal care.

## Introduction

### Problem description

Infants admitted to neonatal intensive care units (NICUs) often require ventilator support as a life-saving intervention. However, unplanned extubation (UE), necessitating reintubation, is the fourth most common adverse event (AE) among NICU patients.^1^

### Available knowledge

The literature offers various definitions of UE, leading to inconsistencies in its characterization and challenges in comparing and generalizing interventions across institutions.^2–5^ Epidemiologic studies have identified multiple risk factors for UE, including an infant’s chronological age, procedural and transport activities, and the type of securement device used.^6^ For example, anatomical and developmental factors, such as the small size of the neonatal trachea (2–6 cm), increase the risk of endotracheal tube (ETT) dislodgment during routine care or procedures.^7^ Additionally, the use of short, uncuffed ETTs exacerbates instability compared to longer, cuffed ETTs.

UE is associated with serious complications, including severe cardiorespiratory events, increased oxygen requirements, post-reintubation sepsis, prolonged NICU stays, higher healthcare costs, and even death prior to discharge.^8–10^ As a result, UE has been nationally recognized as a preventable hospital-acquired condition. In recent years, standardized bundles and validated practices have enabled a few institutions to achieve UE rates below 1.0 per 100 non-tracheostomy ventilation days.^11–14^

### Specific aims

This quality improvement (QI) project aimed to reduce UE events as part of the institutional “zero harm” patient safety initiative. The multidisciplinary Neonatal Quality Council (NQC) was tasked with reducing the baseline UE rate of 1.7 per 100 non-tracheostomy ventilation days to below 1.0 event per 100 non-tracheostomy ventilation days within 12 months.

## Methods

### Context

Cleveland Clinic Children’s NICU provides services across three locations, comprising two Level III units and one Level IV unit, with a total capacity of 87 beds. The Obstetrics department facilitates over 10,000 deliveries annually, resulting in approximately 1300 NICU admissions, including an average of 140 very-low-birth-weight infants (<1500 g) each year. All infants are inborn with <10% transport rate between NICUs within the system. Care across all three NICUs is delivered by the same team of caregivers, following standardized guidelines. Importantly, the guidelines for intubation, mechanical ventilation, and extubation remained unchanged throughout the duration of this project.

### Definitions

At our institution, UE is defined as the unintentional dislodgement of an ETT from the trachea. This definition aligns with the operational criteria established by the Children’s Hospitals’ Solutions for Patient Safety (SPS) network.^15^

### Data collection

Data on non-tracheostomy ventilation days, UE events, and reintubations were extracted from electronic medical records by respiratory team supervisors and stored in a secure institutional drive using Excel spreadsheets.

### Interventions

**Fig. 1:**
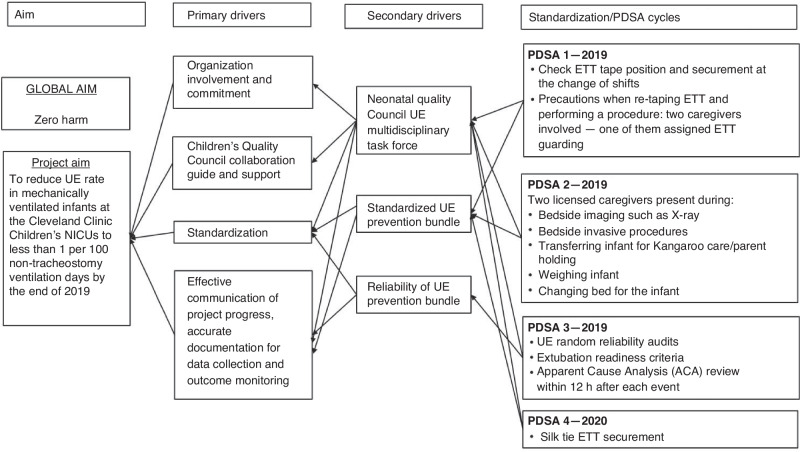
Key driver diagram (KDD) for unplanned extubation. UE unplanned extubation, PDSA plan, do, study, act, ETT endotracheal tube.

The NQC conducted a cause-effect analysis to identify key drivers of UE and developed a UE preventive strategy (Fig. 1). Plan–Do–Study–Act (PDSA) cycles were utilized to implement interventions, with each subsequent cycle informed by the data and findings of the previous cycle.

**Fig. 2:**
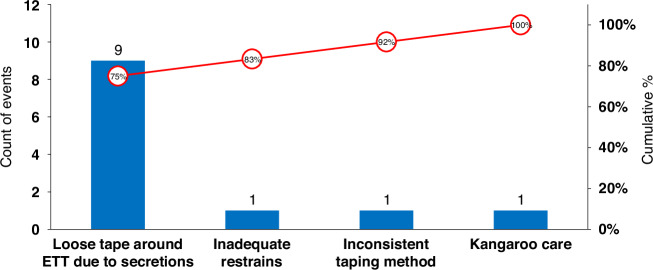
Pareto analysis of major contributing factors for unplanned extubation. ETT unplanned extubation. A total of 12 events were reported. In addition to the major issue of loose taping around ETT, three events occurred during bedside procedures.

- PDSA Cycle 1—January 2019: Focused on evaluating ETT tape position and securement during shift changes. Respiratory therapists emphasized precautions during ETT retaping, requiring the presence of two licensed caregivers: one to stabilize the infant’s head and maintain swaddling and the other to handle the ETT securement.
- PDSA Cycle 2—August 2019: Targeted high-risk situations such as bedside imaging, invasive procedures, kangaroo care transfers, weighing, and bed changes. These activities required the presence of two licensed caregivers.
- PDSA Cycle 3—May 2020: Introduced random chest radiograph (CXR) audits and an apparent cause analysis (ACA) evaluation within 12 h of each UE event across all NICU sites. ACA form is provided in the Supplemental File.
- PDSA Cycle 4—October 2020: Addressed ETT slippage through a modification of the securement method using a silk suture tie in the chevron loop to enhance ETT stability. Routine bedside care included checking tape integrity, but loose tape around the ETT, not easily visible, necessitated this intervention. Illustrations of the silk tie securement method are shown in Fig. 2. Details of this securement method is provided in the Supplemental File. Before implementation, an instructional video was created and made available to staff. All respiratory therapists were required to demonstrate proficiency in applying the silk tie on a mannequin before participating in the actual implementation. Silk tie training was also incorporated into the orientation materials for new respiratory therapists, and a refresher simulation was provided to existing therapists 1 year after implementation. Although physicians and nurses were not required to perform the silk tie procedure, they received orientation on the technique to ensure they could assist respiratory therapists when needed.

The project emphasized a “team buy-in” strategy through active engagement at all levels. To foster open communication, the NQC held staff meetings before the project launch and ahead of each PDSA cycle. Performance metrics were reviewed quarterly, and lessons from UE incidents were shared as “safety stories” during staff meetings. Regular discussions on ETT positioning during grand rounds and other educational activities further reinforced shared ownership of the project.

The QI airway team met every 2 weeks, with leadership receiving monthly updates on the UE rate. Staff were updated on UE rates and engaged in discussions at least quarterly or whenever changes were planned. NICU huddles were conducted after each event to review and address concerns. Before each PDSA cycle, staff were informed during a meeting, and any required training was provided to the involved team members.

### Measures

The primary outcome measure was the UE rate, defined as the number of UE events per 100 non-tracheostomy ventilation days.^15^ The process measures included (a) twice-daily assessment of ETT position and securement by two bedside nurses during shift changes. Respiratory therapists independently performed their checks and corrections each shift; (b) adherence to post-UE event evaluations and completion of the ACA form; and (c) monthly audits of 10 CXRs conducted by respiratory supervisors to ensure proper ETT positioning. Suboptimal CXRs, improper ETT positioning, or missing depth annotations prompted immediate notification of the attending neonatologist for corrective action.

### Balancing measures

These included self-reported difficulties during ETT tape removal, skin integrity after tape removal, and delays in emergent ETT removal due to the use of silk suture ties.

### ACA process

**Fig. 3:**
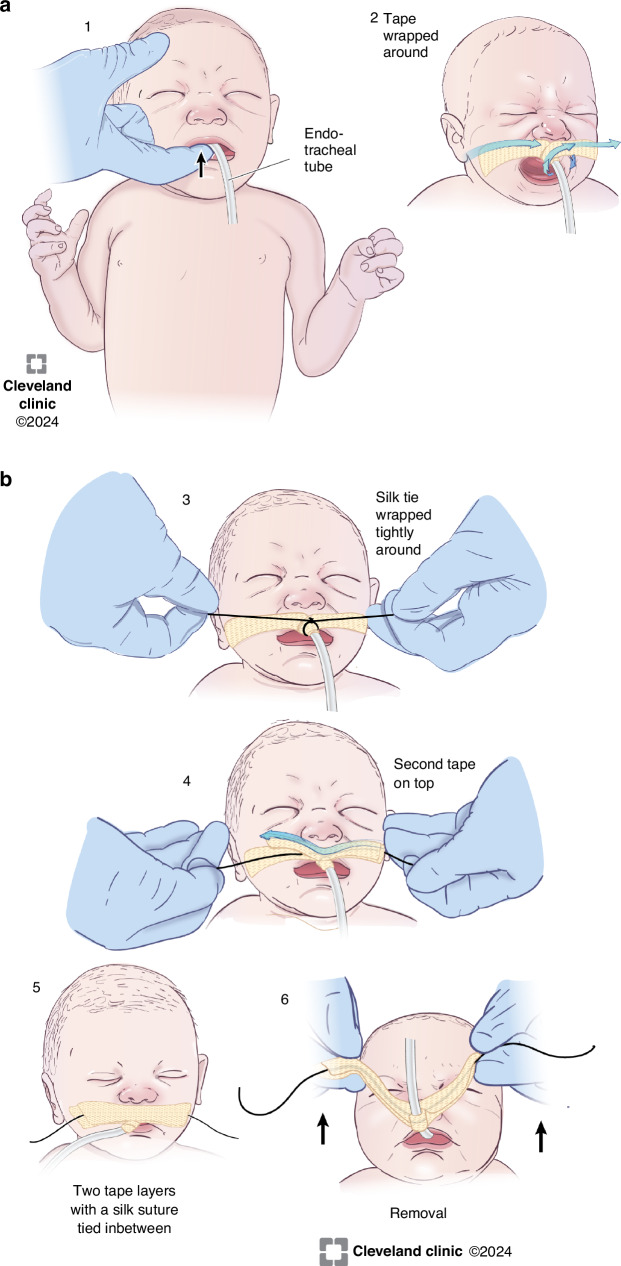
Endotracheal taping and removal using silk tie reinforcement. The details of tube securement are provided in the Supplemental File.

The ACA form, aligned with the SPS reporting format, was implemented in 2020.^16^ It documented AEs related to UE, including arrhythmias, blood pressure changes requiring intervention, increased respiratory support, bradycardia (heart rate <60 bpm), chest compressions, administration of code medications, and delays in ETT removal due to the silk suture tie. Following each UE event, a post-event huddle was conducted within 12 h, during which the ACA form was completed and submitted to the respiratory supervisor. The supervisor presented a summary of findings, contributing factors, and proposed action plans to the NQC-NICU Airway Management Committee. These forms were securely stored, and a Pareto analysis of all UE events in 2020 informed subsequent interventions (Fig. 3).

### Analysis

**Fig. 4:**
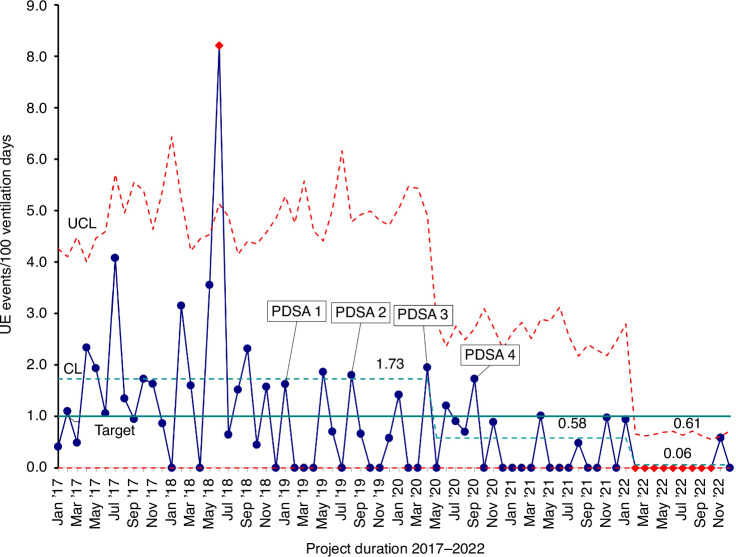
U-Chart to evaluate the efficacy of the intervention. The *X* axis denotes the dates during this QI project, scaled monthly. The *Y* axis represents unplanned extubation events per 100 non-tracheostomy ventilation days. The green dashed line represents the center line (CL), and the red fine dotted line represents the upper control limit (UCL), whereas the green solid line represents the target goal for the QI project (<1 event per 100 ventilation days). UE unplanned extubation, PDSA plan, do, study, act.

The Continuous Improvement Institute at the enterprise dedicated an expert staff (D.K.) who performed all analyses. Analyses were performed using Excel with QI Macros add-on (*QI Macros for Excel*. Version 2025.01. KDT Enterprises, Inc., 2025; https://www.qimacros.com) (Fig. 4). Special cause variations were identified using Nelson’s rules and used to assess process stability. The U-chart, plotted monthly, provided insights into variability, with favorable special cause variations indicating process improvement.

## Results

The characteristics of the NICU population during this period are detailed in Table 1. The baseline UE rate in the years 2017–2018—preceding the project—was 1.73 per 100 non-tracheostomy ventilator days. Following the first three PDSA cycles, the UE rate decreased to 0.88 events per 100 ventilator days; however, this reduction represented a common cause variation, as it did not fulfill the control chart criterion of 8 consecutive points below the center line, and thus did not indicate a statistically significant improvement.

**Table 1:** Demographic and clinical characteristics (*n* = 889).

*Baseline characteristics*	
Gestational age^a^	28 ± 3
Birth weight^a^	1019 ± 332
Female infants	455 (51)
Race	
White	341 (38)
Black	228 (26)
Asian	16 (2)
Singleton	665 (75)
Small for gestational age	184 (21)
Apgar score at 1 min^b^	5 (2–7)
Apgar score at 5 min^b^	7 (5–8)
*Hospital course*	
Surfactant	467 (53)
Bubble continuous positive airway pressure days^a^	13 ± 18
Mechanical ventilation days^a^	9 ± 24
High frequency oscillator ventilation days^a^	2 ± 7
Nitric oxide	81 (9)
Length of stay (days)^b^	57 (34–93)
*Clinical characteristics*	
Apnea of prematurity	793 (90)
Sepsis	217 (24)
Patent ductus arteriosus	250 (28)
Intraventricular hemorrhage (Grades III and IV)	55 (6)
Retinopathy of prematurity (Stage >2)	47 (5)
*Unplanned extubation consequences (n = 30)*	
Need for reintubation	
In 1 h	18 (60)
In 48 h	4 (13)
Chest compression	5 (17)
Epinephrine	1 (3)
Bradycardia (<60 bpm)	9 (30)
Other arrythmia	1 (3)
Escalation of respiratory support	1 (3)

All data are expressed as *n* (%), except ^a^where data are expressed as mean ± standard deviation and ^b^where data are shown in median (interquartile range).

Following the implementation of interventions in PDSA cycle 3 and PDSA cycle 4 (the silk tie), the UE rate further decreased to 0.58 per 100 non-tracheostomy ventilator days. This reduction was considered significant, as the rate remained consistently below the center line for 8 consecutive months.

During the sustainment phase following the four PDSA cycles, the UE rate dropped to zero for 9 consecutive months, with an overall rate of 0.06 per 100 non-tracheostomy ventilator days throughout 2022 (Fig. 4). Although no new formal intervention was introduced at the time of the second center line shift, the improvement was attributed to the cumulative impact of earlier interventions, as well as increased staff experience and proficiency with the silk tie securing method. As familiarity with the technique improved, greater consistency in ETT care likely contributed to the observed special cause variation and sustained reduction in UE rates. Following the completion of this QI project by the end of 2022, UE rate remained within range in 2023, 2024, and 2025 (year to date) with UE rates of 0.11, 0.23, and zero events per 100 non-tracheostomy ventilation days, respectively.

Notably, prior to PDSA cycle 4 in 2020, all UE events were attributed to loose tape. After implementing the silk tie intervention, only two events related to loose tape were recorded—one in 2021 and one in 2022.

Adherence to post-UE event evaluations and completion of the ACA form consistently exceeded 80%, as did compliance with obtaining optimal CXRs for ETT positioning. Importantly, there were no reported delays in ETT removal due to the silk suture tie throughout the intervention period.

Additionally, no other adverse outcomes or delays in resuscitation were attributed to the use of the silk suture tie. No changes in respiratory care unrelated to the interventions were noted or reported during this time that could have influenced the observed outcomes.

With approximately 2000 non-tracheostomy ventilation days annually, this QI initiative reduced UEs from 36 to 1 event, preventing an estimated 35 events per year.

## Discussion

### Summary and interpretation

This QI project achieved the lowest reported UE rates in neonates, sustained for over 2 years. Implementation of four PDSA cycles resulted in eight consecutive points below the mean UE rate, with a significant reduction from 1.725 to 0.58 per 100 ventilator days. The subsequent reduction to 0.06 in February 2022 further validated the sustainability of these interventions. Previous QI projects reported UE rates as low as 0.99 to 0.68 per 100 ventilator days,^11–14^ highlighting the impact of our approach in achieving even greater reductions.

Critical to the project’s success was adherence to QI principles, including fostering team buy-in and active engagement at all levels within the division. Establishing a focused task force, engaging leadership, and adopting a standardized operational definition of UE were foundational steps. Data transparency and regular feedback to frontline caregivers through electronic visual boards, division meetings, regional operational meetings, and huddles effectively motivated staff. Empowering the respiratory team to implement the silk tie intervention in the PDSA cycle 4 further enhanced engagement. Unlike previous studies that required suturing through the ETT wall for stabilization,^17,18^ the current method of securing the ETT with a silk tie avoided the need for needles, streamlining the intervention while maintaining effectiveness. Although a learning curve was present, ongoing education from respiratory supervisors and transparent feedback facilitated widespread adoption.

This QI initiative was well-received by staff, with progress and outcomes attributed to the project fostering enthusiasm across teams. The success of the intervention even motivated colleagues in pediatric critical care units to adopt the approach. It is important to note that the interventions in the four PDSA cycles were not presented as a bundle, as they lacked strong evidence-based literature to support such a framework. Respiratory therapists and bedside nurses were required to document the ETT position and assess securement quality during routine handoff at shift changes. These new practice guidelines were adopted by both nursing and respiratory therapy teams, with full compliance expected. This practice change played a key role in the project’s success. In recent years, standardization of care during high-risk situations and accurate ETT placement have been associated with reduced UE rates.^11–14^ During PDSA cycles 1–3, adopting standardized practices reduced the UE rate to less than 1 per 100 non-tracheostomy ventilation days by early 2020. This reproducible effect stemmed from minimizing variations in patient care and eliminating special cause variations, creating a highly reliable and stable system. The stability allowed for precise identification of the most significant contributors to UE, leading to the PDSA cycle 4 intervention that addressed ETT slippage through the wet chevron loop. Standardizing the definition of UE events was also critical for comparing institutional outcomes to benchmarks. At our institution, the UE definition aligned with that used for the SPS Network, ensuring consistency in reporting and data accuracy.

UEs and subsequent reintubations are associated with AEs, including bradycardia, oxygen desaturation, airway trauma, prolonged ventilator support, and extended hospital stays. Serious events such as chest compressions, epinephrine administration, or even death have been reported.^8,19^ By reducing the UE rate to less than 1 per 100 ventilator days, as seen in other institutions,^20–22^ this project minimized these risks. Further reductions to an average UE rate of 0.06 in 2022 highlight the silk tie intervention as a positive special cause variation, enabling a stable and reliable system that is ripe for future improvements.

Addressing the “rising tide phenomenon” is important for interpreting results. System-wide improvements often occur once healthcare teams recognize an issue, narrowing the observed differences between control and intervention groups in prospective studies.^23^ Acknowledging this phenomenon is essential to accurately interpret outcomes and avoid overgeneralizing findings to other healthcare systems where such trends may not exist. In the context of this project, the improvement in the UE rate was not immediate. Significant improvement occurred after PDSA cycle 4, making the influence of the rising tide phenomenon unlikely.

### Strengths and limitations

The silk tie intervention was easily adopted following a single demonstration on a mannequin in each NICU. This process change did not require additional securement devices or costs, making it feasible for low-resource settings, including low- and middle-income countries. Additional benefits included reduced exposure to unnecessary reintubations, decreased facial skin compromise from repetitive retaping, and improved caregiver and parental satisfaction, as reported anecdotally during staff meetings. The silk tie was not associated with skin breakdown, as the tie is positioned between two layers of tape without direct contact with the skin. When the decision is made to remove the tube, the silk tie does not cause delays since the entire tube, tape, and silk tie are removed together. However, if retaping the tube is required, the process can be time-consuming. In such cases, two caregivers are involved: one carefully removes the superficial layer of tape, cuts the silk tie, removes the deep layer of tape, adjusts the tube position, and then retapes the tube securely.

However, there are limitations to this initiative. Standardized data on pain scores, ETT taping-related skin breakdown, and caregiver or parental satisfaction were not collected. While the intervention was implemented in three NICUs with different respiratory therapists and nursing teams, all belonged to a single enterprise with one physicians’ group, limiting the generalizability of these findings. Additionally, the sustained reduction in UE rates to 0.06 in 2022 may partly reflect growing experience and skill with the silk tie technique, though no objective measures of this were captured. Greater involvement of parents and families in the NICU could have provided valuable feedback and enhanced the initiative.

## Conclusions

This QI project demonstrates that achieving the lowest reported UE rates in neonatal ICUs is possible without incurring additional costs or requiring new securement devices. The success of this project underscores the importance of standardizing care, reducing variability, and fostering a multidisciplinary, collaborative approach. Once ETT securement procedures and UE definitions are standardized, additional interventions can be introduced to further optimize outcomes. The silk tie enhancement for ETT stabilization emerged as the most impactful intervention in reducing UEs, supported by process improvements such as CXR audits and ACA implementation. While the earlier steps contributed to optimizing care practices, sustained low UE rates have been primarily attributed to interventions introduced in PDSA cycles 3 and 4.

## Supplementary information

Supplement Figure

Supplement

## Data Availability

The dataset generated during and/or analyzed during the current study is available from the corresponding author on reasonable request.
