# Supplementary material for: Prevention of unplanned extubation in neonates with silk tie securement
Source: Pediatr Res. 2025 Jun 12;99(2):527–33. doi: 10.1038/s41390-025-04168-w (PMC12956563; doi:10.1038/s41390-025-04168-w)
Supplement: Supplementary file 1 — Supplement Figure [file 41390_2025_4168_MOESM1_ESM.pdf]

Apparent Cause Analysis (RCA) Form

Patient Label

Date of Event: ________________ Time of Event: ________________ Location: □ M43 □ M53 □ M31

History of Prior Unplanned Extubation:

□ Yes □ No

Airway Prior to Unplanned Extubation:

□ Nasal □ Oral HFOV □ Yes □ No

□ Uncuffed □ Cuffed – if checked □ Cuff inflated □ Cuff deflated

Oral ETT location □ Corner of mouth □ Center of mouth

In-line (closed) suction apparatus attached □ Yes □ No

Phase of Treatment:

□ Acute – Care is escalating or static

□ Weaning – Patient is improving and vent settings are de-escalating

□ Awaiting extubation

Ordered Sedation Prior to Unplanned Extubation (may check multiple):

□ Continuous □ Scheduled

□ PRN □ None

Staffing:

Nursing assignment □ 1:1 □ 1:2 RT to vent ratio _______

Nurse present in room at time of unplanned extubation □ Yes □ No

# of individuals in room at time of unplanned extubation _______

Did the Patient Require Reintubation:

Within 1 hr □ Yes □ No Within 48 hr □ Yes □ No

Chest compression requirement during reintubation □ Yes □ No

Narrative (prior sedation, events, etc.):

Contributors to Unplanned Extubation:

	Yes	No
Inadequate Restraints		
Loose Tape – On Face		
Loose Tape – On ETT		
Inadequate Sedation		
Improper Staffing Handoff		
Within 1 hour of Admission		
Other		

Activities Occurring at Time of Unplanned Extubation:

	Yes	No
Kangaroo Care or Parent Holding		
Radiology Study (xray, US, echo, etc.)		
Respiratory Treatment		
ETT adjustment/Retaping ETT		
Weighing Patient		
Position Change		
Transport		
Inline Suctioning		
Open Suctioning		
Excessive Secretions Present		
Patient Coughing		
Bathing		
Line Placement		
Blood Draw		
Other Bedside Procedure		

Adverse Events Resulting From Unplanned Extubation:

	Yes	No
Arrhythmia		
BP Change Requiring Intervention		
Increased Respiratory Modalities		
Heart Rate <60		
Chest Compressions		
Code Medications Given		

MD/NP ________________________________ RT ________________________________ RN ________________________________

*** Not Part of the Patient's Medical Record***
