# Supplementary material for: Prevention of unplanned extubation in neonates with silk tie securement
Source: Pediatr Res. 2025 Jun 12;99(2):527–33. doi: 10.1038/s41390-025-04168-w (PMC12956563; doi:10.1038/s41390-025-04168-w)
Supplement: Supplementary file 2 — Supplement [file 41390_2025_4168_MOESM2_ESM.pdf]

Conventional method of the ETT securement:

The standard procedure for the ETT securement across the Cleveland Clinic Children's Hospital NICU sites involved applying two layers of tape in the chevron loop around the ETT, with a third layer securing the first two. Uncuffed ETT was predominantly used in the NICU. However, for better monitoring ventilation in the operation room, surgical patients were intubated with cuffed tubes.

Modified method of the ETT securement with a silk suture tie:

The ETT is first secured to the infant's face with a strip of tape in the chevron loop. Subsequently, a 2.0 silk suture is triple-knotted over the initial layer of tape and then single-knotted for additional security (Fig. 4). The suture lengths are spread across the tape on the lip to avoid direct contact with the face. The second chevron loop tape is then placed over the suture to sandwich it between the first and second layers. The taping is finalized with a third layer securing the initial two. Figure 4 demonstrates the steps for suture application and for the emergent removal of the ETT when needed.

For the initial intubation in delivery room, all ETTs are secured by the conventional method. Once the patient is stabilized in the NICU and ETT placement is confirmed radiographically, the second chevron loop is removed, and the silk suture tie is applied as described above.

The application of the silk suture tie has no interference with the emergent ETT removal as the tube can be removed with the tape in one step altogether. If the tube depth needs adjustment based on recent chest radiograph, the tape and the silk tie will be removed and a fish tape and silk tie will be used.

Education and implementation of the modified method of the ETT securement:

The intervention, along with an educational package, received approval from the Neonatal Executive Committee in September 2020. This educational package was subsequently presented during the general staff meeting and reiterated at the operations meeting at each NICU site. Bedside nurses received a flyer with needed instructions. Respiratory therapists (RTs) were provided with a detailed instructional video on the application of silk suture tie to the endotracheal tube (ETT). Subsequently, proctored hands-on training using mannequins were fulfilled to refine the technique. The intervention was implemented across all NICU facilities in September 2020.
